# Transient Knock-Down of Prefrontal DISC1 in Immune-Challenged Mice Causes Abnormal Long-Range Coupling and Cognitive Dysfunction throughout Development

**DOI:** 10.1523/JNEUROSCI.2170-18.2018

**Published:** 2019-02-13

**Authors:** Xiaxia Xu, Mattia Chini, Sebastian H. Bitzenhofer, Ileana L. Hanganu-Opatz

**Affiliations:** Developmental Neurophysiology, Institute of Neuroanatomy, University Medical Center Hamburg-Eppendorf, 20251 Hamburg, Germany

**Keywords:** development, DISC1, network oscillations, prefrontal maturation, prefrontal–hippocampal communication

## Abstract

Compromised brain development has been hypothesized to account for mental illness. This concept was underpinned by the function of the molecule disrupted-in-schizophrenia 1 (DISC1), which represents an intracellular hub of developmental processes and has been related to cognitive dysfunction in psychiatric disorders. Mice with whole-brain DISC1 knock-down show impaired prefrontal–hippocampal function and cognitive abilities throughout development and at adulthood, especially when combined with early environmental stressors, such as maternal immune activation (MIA). However, the contribution of abnormal DISC1-driven maturation of either prefrontal cortex (PFC) or hippocampus (HP) to these deficits is still unknown. Here, we use *in utero* electroporation to restrict the DISC1 knock-down to prefrontal layer II/III pyramidal neurons during perinatal development and expose these mice to MIA as an environmental stressor (dual-hit G_PFC_E mice, both sexes). Combining *in vivo* electrophysiology and neuroanatomy with behavioral testing, we show that G_PFC_E mice at neonatal age have abnormal patterns of oscillatory activity and firing in PFC, but not HP. Abnormal firing rates in PFC of G_PFC_E mice relate to sparser dendritic arborization and lower spine density. Moreover, the long-range coupling within prefrontal–hippocampal networks is decreased at this age. The transient prefrontal DISC1 knock-down was sufficient to permanently perturb the prefrontal–hippocampal communication and caused poorer recognition memory performance at pre-juvenile age. Thus, developmental dysfunction of prefrontal circuitry causes long-lasting disturbances related to mental illness.

**SIGNIFICANCE STATEMENT** Hypofrontality is considered a main cause of cognitive deficits in mental disorders, yet the underlying mechanisms are still largely unknown. During development, long before the emergence of disease symptoms, the functional coupling within the prefrontal–hippocampal network, which is the core brain circuit involved in cognitive processing, is reduced. To assess to which extent impaired prefrontal development contributes to the early dysfunction, immune-challenged mice with transient DISC1 knock-down confined to PFC were investigated in their prefrontal–hippocampal communication throughout development by *in vivo* electrophysiology and behavioral testing. We show that perturbing developmental processes of prefrontal layer II/III pyramidal neurons is sufficient to diminish prefrontal–hippocampal coupling and decrease the cognitive performance throughout development.

## Introduction

The cerebral cortex emerges as the result of complex developmental processes, such as neurogenesis, neuronal migration, and differentiation (Rakic, 1988; Dehay and Kennedy, 2007). They are controlled by numerous cell autonomous process as well as extracellular and environmental factors. Disrupted-in-schizophrenia 1 (DISC1) is an intracellular scaffold protein that has been identified as an intracellular hub of developmental processes (Narayan et al., 2013). Moreover, DISC1 plays a critical role for synapse regulation. Despite its name, which reflects a unique finding of a familial aggregation of major mental illness (Millar et al., 2000), according to recent investigations, DISC1 is unlikely to be a “genetic” factor causing schizophrenia (Ripke et al., 2013; Sullivan, 2013). Instead, DISC1 points out the relevance of abnormal development in multiple mental conditions, because it orchestrates molecular cascades hypothesized to underlie disease-relevant physiological and behavioral abnormalities (Cuthbert and Insel, 2013). Dysfunction of DISC1 mimicked in several mouse models led to cellular, neurotransmitter, circuitry, and behavioral deficits at adulthood (Tomoda et al., 2016). In particular, the disruption of limbic circuits centered on the prefrontal–hippocampal networks and the impairment of memory and executive abilities have been previously reported in DISC1 haploinsufficiency, transgenic and point mutation models, as well as in models mimicking the additional disruption of *Disc1* locus by environmental stressors (Koike et al., 2006; Clapcote et al., 2007; Kvajo et al., 2011; Jaaro-Peled et al., 2013; Niwa et al., 2013; Crabtree et al., 2017). Whereas the initial alteration of developmental molecular cascades controlled by DISC1 and its final readout at physiological and behavioral level have been largely elucidated, the patterns of circuit miswiring during early development in mice with DISC1 dysfunction are still poorly understood.

Recent findings showed that the prefrontal and hippocampal circuits are tightly linked throughout development (Brockmann et al., 2011). Shortly after birth, the prefrontal cortex (PFC) starts to generate coordinated patterns of oscillatory activity that results both from the entrainment of local circuits and the driving force of theta oscillations in the intermediate/ventral hippocampus (HP; Bitzenhofer et al., 2017b; Ahlbeck et al., 2018). At this age, the monosynaptic projections from CA1 pyramidal neurons target the deep layers of prelimbic subdivision (PL) of PFC, whereas no direct feedback connectivity exists. The unidirectional drive from HP to PL via axonal projections is maintained also at adulthood (Thierry et al., 2000) and controls memory and executive performance. For example, temporal coordination of prefrontal ensembles by hippocampal oscillatory rhythms is critical for different memory forms (Siapas et al., 2005; Fujisawa and Buzsáki, 2011; Spellman et al., 2015; Backus et al., 2016).

DISC1 dysfunction perturbs not only the adult prefrontal–hippocampal coupling but also its maturation. We previously found that, in comparison with control mice, the drive from HP to PL is weaker at neonatal age and augmented at pre-juvenile age in prenatally immune challenged mice containing a whole-brain truncated form of DISC1 (Hartung et al., 2016b). Several mechanisms may account for these communication deficits: (1) DISC1-controlled abnormal maturation of PFC is critical, (2) DISC1-controlled maturation of HP is critical, (3) abnormal development of both areas as a result of DISC1 deregulation is necessary, and finally, (4) DISC1 deficiency causes aberrant connectivity from HP to PFC. “Here, we test the first mechanism aiming to elucidate whether DISC1-controlled developmental deficits confined to PFC lead to similar impairment of prefrontal–hippocampal communication as previously reported for whole-brain deregulation of DISC1”. Because our previous data showed that at neonatal age DISC1 dysfunction is not sufficient to perturb the prefrontal–hippocampal activity and coupling, the abnormal genetic background (one-hit G) was combined with an environmental stressor (i.e., maternal immune activation, one-hit E). We used *in utero* electroporation (IUE) to selectively knock down DISC1 in prefrontal layer II/III pyramidal neurons during perinatal development in mice exposed to maternal immune activation (MIA) as environmental stressor (dual-hit G_PFC_E mice). We combine *in vivo* electrophysiology with behavioral assessment to elucidate the deficits of dual-hit G_PFC_E mice throughout development.

## Materials and Methods

Experiments were performed in compliance with the German laws and the guidelines of the European Community for the use of animals in research and were approved by the local ethical committee (111/12, 132/12). Timed-pregnant C57BL/6J mice from the animal facility of the University Medical Center Hamburg-Eppendorf were used. The day of vaginal plug detection was defined as embryonic day (E)0.5, whereas the day of birth was defined as postnatal day (P)0.

### 

#### Experimental design

**Figure 1.**
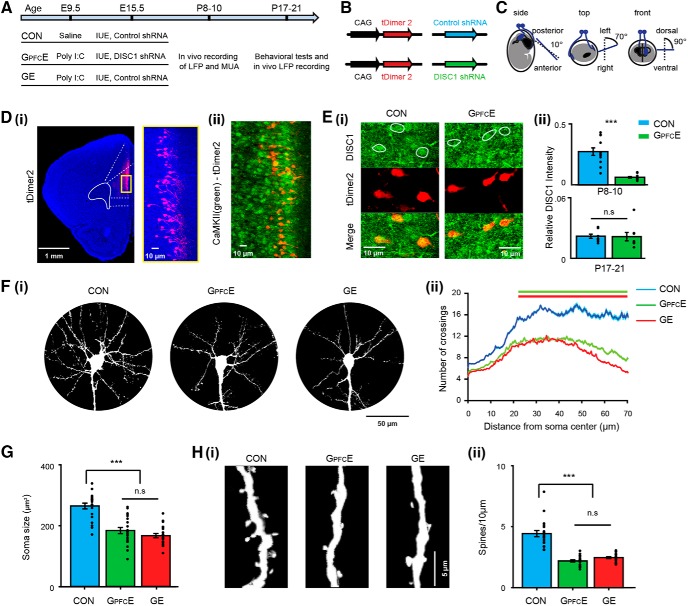
Transient DISC1 knock-down confined to pyramidal neurons in PFC by site-directed IUE. ***A***, Timeline of experimental protocol and description of the three investigated groups of mice: CON, immune-challenged mice with transient suppression of DISC1 confined to PFC (G_PFC_E), and immune-challenged mice with brain-wide DISC1 knock-down. ***B***, Structure of the constructs. ***C***, Schematic drawing illustrating the orientation of electrode paddles for specific targeting of pyramidal neurons in layer II/III of PFC by IUE. ***Di***, tDimer2-expressing cells (red) in a 50-μm-thick coronal section including the PL from a P9 mouse after IUE at E15.5. Inset, Photograph displaying the targeted neurons at higher-magnification. ***Dii***, Photograph displaying CaMKII immunohistochemistry (green) in relationship to tDimer2-expression (red). ***Ei***, Photographs displaying DISC1 immunoreactivity (green) in relationship with tDimer2-expression (red) of a P9 G_PFC_E mice compared with age-matched CON (one-way ANOVA: *p* = 0.0000, *F*_(1,23)_ = 48.07). ***Eii***, Bar diagram displaying the relative DISC1 immunoreactivity averaged for G_PFC_E and CON mice at P8–P10 (top) and P17–P21 (bottom; one-way ANOVA: *p* = 0.47, *F*_(1,14)_ = 4.07). ***Fi***, Photographs of representative layer II/III pyramidal neurons in a P9 CON, a P9 G_PFC_E, and a P9 GE mouse. ***Fii***, Graph displaying the average number of dendritic intersections within a 70 μm radius from the soma center of layer II/III pyramidal neurons in CON (*n* = 21 neurons from 3 mice), G_PFC_E (*n* = 21 neurons from 4 mice), and GE (*n* = 21 neurons from 3 mice) mice. Green and red bars indicate significant differences (***p < 0.001) between CON and G_PFC_E mice and between CON and GE mice, respectively. ***G***, Bar diagram displaying the soma size of prefrontal layer II/III pyramidal neurons in CON, G_PFC_E, and GE mice. ***Hi***, Photograph displaying representative apical dendrites of a prefrontal layer II/III pyramidal neuron from a P9 CON, a P9 G_PFC_E, and a P9 GE mouse. ***Hii***, Bar diagram displaying spine density on dendrites of prefrontal layer II/III pyramidal neurons from CON (20 neurons from 3 mice), G_PFC_E (21 neurons from 3 mice) and GE (21 neurons from 3 mice; one-way ANOVA: *p* = 0.47, *F*_(2,59)_ = 59.43). Data are presented as mean ± SEM. Significance levels of *p* > 0.05 (n.s.), *p* < 0.001 (***) were detected.

Mice were transfected with either (1) short-hairpin RNA (shRNA) to DISC1 (5′-GGCAAACACTGTGAAGTGC-3′) to selectively knock down the expression of DISC1 in PFC during neonatal development (Niwa et al., 2010) or (2) scrambled target sequence without homology to any known messenger RNA (5′-ATCTCGCTTGGGCGAGAGT-3′) as control shRNA. Both shRNA to DISC1 and control shRNA were expressed under H1 promoter-driven pSuper plasmid. To visualize the transfected neurons, DISC1 shRNA or control shRNA was expressed together with tDimer2 under the control of the CAG promoter (pAAV-CAG-tDimer2). Three groups of mice were investigated. First, the offspring of pregnant wild-type C57BL/6J dams, which were injected at gestational day (G)9.5 with the viral mimetic poly I:C (4 mg/kg, i.v.), were transfected by IUE with DISC1 shRNA at E15.5. These mice mimicking the dual genetic and environmental (i.e., MIA) etiology of disease were classified as G_PFC_E mice. Second, the heterozygous offspring of pregnant dams carrying a DISC1 allele (DISC1^Tm1Kara^) on a C57BL/6J background and injected at E9.5 with the viral mimetic poly I:C (4 mg/kg, i.v.) were transfected by IUE with control shRNA at E15.5 and classified as dual-hit genetic-environmental (GE) mice. Third, the offspring of pregnant wild-type C57BL/6J dams injected at E9.5 with saline (0.9%, i.v) were transfected with control shRNA and were classified as controls (CON; Fig. 1*A*,*B*). Multisite extracellular recordings and behavioral testing were performed on pups of both sexes during neonatal development at P8–P10 as well as during pre-juvenile development at P16–P23 (Fig. 1*A*).

#### *In utero* electroporation

The transfection of prefrontal neurons with the constructs indicated above was performed according to previously developed protocols (Bitzenhofer et al., 2017a,b; Ahlbeck et al., 2018). Starting 1 d before and until 2 d after surgery, timed-pregnant C57BL/6J mice received on a daily basis additional wet food supplemented with 2–4 drops Metacam (0.5 mg/ml; Boehringer-Ingelheim). At E15.5 randomly assigned pregnant mice were injected subcutaneously with buprenorphine (0.05 mg/kg body weight) 30 min before surgery. The surgery was performed on a heating blanket and toe pinch and breathing were monitored throughout. Under isoflurane anesthesia (induction: 5%, maintenance: 3.5%), the eyes of the dam were covered with eye ointment to prevent damage. The uterine horns were exposed and moistened with warm sterile PBS (PBS, 37°C). Solution containing shRNA to DISC1 or control RNA plasmids (1.5 mg/ml) together with the tDimer expression vector with CAG promoter (1 mg/ml; molar ratio ∼3:1) were injected into the right ventricles of individual embryo using pulled borosilicate glass capillaries. Injected solution contained fast green solution (0.001%) to monitor the injection. After the injection, the head of the embryo was placed between the electroporation tweezer-type paddles of 5 mm diameter (Protech). To transfect the neural precursor cells from the subventricular zone, electrodes were oriented at a rough 20° leftward angle from the midline and a rough 10° angle downward from anterior to posterior. Five electrode pulses (35 V, 50 ms) at intervals of 950 ms were applied, which were controlled by an electroporator (CU21EX, BEX). After electroporation, uterine horns were put back into the abdominal cavity filled with warm sterile PBS (37°C). The abdominal wall and skin were sutured individually with absorbable and non-absorbable suture threads, respectively. After surgery, pregnant mice were returned to their home cages, which were half placed on a heating blanket for the following 2 d. The tDimer2 expression was first checked by a portable fluorescent flashlight (Nightsea) through the intact skull and skin at P3 and confirmed postmortem by fluorescence microscopy in brain slices at P8–P10 or P17–P23.

#### Electrophysiological recordings *in vivo*

Multisite extracellular recordings were performed in the PL and HP of P8–P10 and P20–P23 pups of both sexes. Mice were injected intraperitoneally with urethane (1 mg/g body weight; Sigma-Aldrich) before surgery. Under isoflurane anesthesia (induction: 5%; maintenance: 2.5%) the head of the pup was fixed into a stereotaxic apparatus using two plastic bars mounted on the nasal and occipital bones with dental cement. The bone over the PFC (0.8 mm anterior to bregma, 0.1–0.5 mm right to the midline) and the CA1 area of the intermediate HP (3.5–3.7 mm anterior to bregma, 3.5–3.8 mm right to the midline) was carefully removed by drilling holes <0.5 mm in diameter. Four-shank electrodes (4 × 4 recording sites, 0.4–0.8 MΩ impedance, 100 mm spacing, 125 mm inter-shank spacing; NeuroNexus) were inserted into PFC at a depth of 1.9 mm from the skull surface. One-shank electrodes (1 × 16 recording sites, 0.4–0.8 MΩ impedance, 50 mm spacing, NeuroNexus) were inserted into the CA1 until a depth of 1.3–1.8 mm from the skull surface, at an angle of 20° from the vertical plane. Electrodes were labeled with DiI (1,1′-dioctadecyl-3,3,3′,3′-tetramethyl indocarbocyanine; Invitrogen) to confirm their position after histological assessment postmortem. In PL, the most medial shank was confirmed to lay into layer II/III, whereas the most lateral shank was located in layer V/VI. In hippocampal CA1 area the LFP reversal over stratum pyramidale was used for the selection of the channel with sharp waves of minimum amplitude and consequently, lowest contribution to the spectral content of the signal. One silver wire was inserted into cerebellum to serve as ground and reference electrode. A recovery period of 10 min following the insertion of electrodes before acquisition of data was provided. Data acquired during the first 30 min of recording were used for analysis to ensure similar state of anesthesia in all investigated pups. Extracellular signals were bandpass filtered (0.1 Hz to 5 kHz) and digitized (32 kHz) with a multichannel extracellular amplifier (Digital Lynx SX, Neuralynx) and the Cheetah acquisition software (Neuralynx).

#### Behavioral experiments

The exploratory behavior and recognition memory of CON, GE, and G_PFC_E mice were tested at pre-juvenile age using previously established experimental protocols (Krüger et al., 2012). Briefly, all behavioral tests were conducted in a circular white arena, the size of which (D: 34 cm, H: 30 cm) maximized exploratory behavior, while minimizing incidental contact with testing objects (Heyser and Ferris, 2013). The objects used for testing of novelty recognition were six differently shaped, textured and colored, easy to clean items that were provided with magnets to fix them to the bottom of the arena. Object sizes (H: 3 cm, diameter: 1.5–3 cm) were smaller than twice the size of the mouse and did not resemble living stimuli (no eye spots, predator shape). The objects were positioned at 10 cm from the borders and 8 cm from the center of the arena. After every trial the objects and arena were cleaned with 0.1% acetic acid to remove all odors. A black and white CCD camera (Videor Technical E. Hartig) was mounted 100 cm above the arena and connected to a PC via PCI interface serving as frame grabber for video tracking software (Video Mot2 software, TSE Systems).

##### Exploratory behavior in the open field.

Pre-juvenile mice (P16) were allowed to freely explore the testing arena for 10 min. Additionally, the floor area of the arena was digitally subdivided in eight zones (4 center zones and 4 border zones) using the zone monitor mode of the VideoMot 2 analysis software (VideoMot 2, TSE Systems). The time spent by pups in center and border zones, as well as the running distance and velocity were quantified.

##### Novelty recognition paradigms.

All protocols for assessing item recognition memory in P17 mice consisted of familiarization and testing trials (Ennaceur and Delacour, 1988). During the familiarization trial each mouse was placed into the arena containing two identical objects and released against the center of the opposite wall with the back to the objects. After 10 min of free exploration of objects the mouse was returned to a temporary holding cage. Subsequently, the test trial was performed after a delay of 5 min post-familiarization. The mice were allowed to investigate one familiar and one novel object with a different shape and texture for 5 min. Object interaction during the first 3 min was analyzed and compared between the groups. In the object location recognition (OLR) task, tested at P18, mice experienced one 10-min-long familiarization trial with two identical objects followed after a delay of 5 min by a test trial. In the test trial the position of one of the objects was changed. Object interaction during the first 3 min was analyzed and compared between the groups. In the recency recognition (RR) task, tested at P19–P20, mice experienced two 10-min-long familiarization trials with two different sets of identical objects that were separated by a delay of 30 min. The second familiarization trial was followed after 5 min by a test trial in which one object used in the first and one object used in the second more recent familiarization trial were placed in the arena at the same positions as during the familiarization trials. Object interaction during the first 3 min was analyzed and compared between the groups. All trials were video-tracked and the analysis was performed using the Video Mot2 analysis software. The object recognition module of the software was used and a three-point tracking method identified the head, the rear end and the center of gravity of the mouse. Digitally, a circular zone of 1.5 cm was created around each object and every entry of the head point into this area was considered as object interaction. Climbing or sitting on the object, mirrored by the presence of both head and center of gravity points within the circular zone, were not counted as interactions.

#### Histology and immunohistochemistry

Histological procedures were performed as previously described (Bitzenhofer et al., 2017b). Briefly, P8–P10 and P20–P23 mice were anesthetized with 10% ketamine (aniMedica)/2% xylazine (WDT) in 0.9% NaCl solution (10 μg/g body weight, i.p.) and transcardially perfused with Histofix (Carl Roth) containing 4% paraformaldehyde. Brains were postfixed with 4% paraformaldehyde for 24 h and sectioned coronally at 50 μm. Free-floating slices were permeabilized and blocked with PBS containing 0.8% Triton X-100 (Sigma-Aldrich), 5% normal bovine serum (Jackson ImmunoResearch) and 0.05% sodium azide. Subsequently, slices were incubated with the rabbit polyclonal primary antibody against CaMKII (1:200; PA5-38239, ThermoFisher Scientific) or against DISC1 (1:250; 40-6800, ThermoFisher Scientific), followed by 2 h incubation with AlexaFluor-488 goat anti-rabbit IgG secondary antibody (1:500; A11008, Merck Millipore). Slices were transferred to glass slides and covered with Fluoromount (Sigma-Aldrich). Wide field fluorescence images were acquired to reconstruct the recording electrode position and the location of tDimer2 expression. High-magnification images were acquired by confocal microscope (DM IRBE, Leica) to quantify DISCI expression (DISC1-immunopositive cells) in tDimer-neurons (3∼4/per slice). All images were similarly analyzed with ImageJ.

#### Neuronal morphological analysis

Microscopic stacks were examined on a confocal microscope (DM IRBE, Leica Microsystems, Zeiss LSN700 and Olympus FX-100). Stacks were acquired as 2048 × 2048 pixel images (pixel size, 78 nm; *Z*-step, 500 nm). Sholl analysis and spine density quantification were performed in the ImageJ environment. For Sholl analysis, images were binarized (*auto threshold*) and dendrites were traced using the semiautomatic plugin *Simple Neurite Tracer*. The traced dendritic tree was analyzed with the plugin *Sholl Analysis*, after the geometric center was identified using the *blow/lasso* tool. For spine density quantification, we first traced the dendrite of interest (apical, basal, proximal oblique, or secondary apical) and measured its length (*line*) and then manually counted the number of spines (*point picker*).

#### Data analysis

Data were imported and analyzed off-line using custom-written tools in MATLAB software version 7.7 (MathWorks). The data were processed as follows: (1) bandpass filtered (500–5000 Hz) to detect multiple unit activity (MUA) as negative deflections exceeding five times the SD of the filtered signals and (2) low-pass filtered (<1500 Hz) using a third-order Butterworth filter before downsampling to 1000 Hz to analyze the LFP. All filtering procedures were performed in a phase-preserving manner. The position of Di-stained recording electrodes in PL (most medial shank confined to layer II/III, most temporal shank confined to layer V/VI) and HP was confirmed postmortem by histological evaluation. Additionally, electrophysiological features (i.e., reversal of LFP and high MUA frequency over stratum pyramidale of CA1) were used for confirmation of exact recording position in HP.

##### Detection of neonatal oscillatory activity.

Discontinuous oscillatory events were detected using a previously developed unsupervised algorithm (Cichon et al., 2014) and confirmed by visual inspection. Briefly, deflections of the root-mean-square of bandpass (3–100 Hz) filtered signals exceeding a variance-depending threshold were assigned as network oscillations. The threshold was determined by a Gaussian fit to the values ranging from 0 to the global maximum of the root-mean-square histogram. Only oscillatory events >1 s were considered for further analysis. Time-frequency plots were calculated by transforming the data using the Morlet continuous wavelet.

##### Detection of sharp waves in HP.

To analyze sharp waves, we subtracted the filtered signal (1–300 Hz) from the recording sites 100 μm above and 100 μm below the recording site located in stratum pyramidale. Sharp waves were detected as peaks >5 times the SD of the subtracted signal.

##### Spectral coherence.

Coherence was calculated using the imaginary coherency method (Nolte et al., 2004). Briefly, the imaginary coherence was calculated (using the functions *cpsd.m* and *pwelch.m*) by taking the imaginary component of the cross-spectral density between the two signals and normalized by the power spectral density of each. were used. The computation of the imaginary coherence *C* over frequency (*f*) for the power spectral density *P* of signals *X* and *Y* was performed according to the following formula:

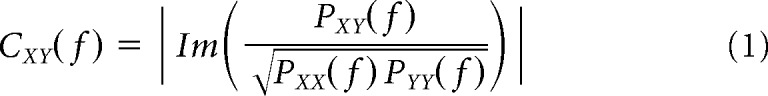


##### Directionality methods.

To investigate the directionality of functional connectivity between PFC and HP, cross-correlation, and generalized partial directed coherence (gPDC) were used. For the calculation of cross-correlation at different time lags, LFP signals from both areas were filtered into theta (4–12 Hz) and β (12–30 Hz) frequency bands. The peak values of cross-correlation and the corresponding time delays were determined. gPDC is based on linear Granger causality measure in the frequency domain. The method attempts to describe the causal relationship between multivariate time series based on the decomposition of multivariate partial coherence computed from multivariate autoregressive models. The LFP signal was divided into 1-s-long segments containing the oscillatory activity. After de-noising using the MATLAB wavelet toolbox, gPDC was calculated using a previously described algorithm (Baccalá and Sameshima, 2001; Baccalá et al., 2007).

##### Spike-triggered LFP power in PFC.

Spiking activity in layers II/III and V/VI was detected as described above. The percentage change of spike-triggered LFP power spectrum was calculated as follows:

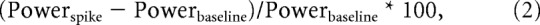
 where Power_spike_ corresponds to the power spectrum calculated for a 200 ms time window centered on each spike and Power_baseline_ stands for the averaged baseline power spectrum calculated 100–300 and 200–400 ms before each spike. Power spectra were calculated using the multitaper spectral estimation method (Mitra and Bokil, 2008).

#### Statistical analysis

Statistical analyses were performed with IBM SPSS Statistics version 21 or MATLAB. Significant differences were detected by paired *t* test or one-way ANOVA followed by Bonferroni-corrected *post hoc* analysis. For Sholl analysis, one-way repeated-measures ANOVA was used. Data are presented as mean ± SEM. Significance levels of *p < 0.05,**p < 0.01, or ***p < 0.001 were tested.

## Results

### Transient DISC1 knock-down confined to layer II/III pyramidal neurons disturbs the firing and oscillatory entrainment in PFC of neonatal immune-challenged mice

To assess the PFC-specific role of DISC1, we generated G_PFC_E mice in which the selective knock-down of DISC1 was restricted to a lineage of pyramidal neurons in PFC. To do so, we expressed a DISC1 targeting shRNA by using IUE protocols previously described (Niwa et al., 2010; Bitzenhofer et al., 2017b). We analyzed coronal sections from three mice at P9 and confirmed that only CaMKII-positive pyramidal neurons in layer II/III were targeted in G_PFC_E mice (Fig. 1*A–D*). Similar IUE protocol was used for CON and GE mice that received a scrambled/control shRNA instead (Fig. 1*A*). The immune challenge of G_PFC_E and GE mice was mimicked by prenatal immune activation with the viral mimetic poly I:C at E9.5. CON mice, instead, received saline injections at the same age. We found that the suppression of DISC1 was transient. When sections containing the PFC of P8–P10 mice were analyzed, the relative DISC1 intensity was significantly (*p* < 0.0001, ANOVA) weaker in G_PFC_E (0.062 ± 0.006, *n* = 13 mice) compared with CON mice (0.275 ± 0.033, *n* = 12 mice; Fig. 1*E*). In contrast, at P17–P21, DISC1 expression was at comparable levels in the two groups of mice (0.021 ± 0.004 in CON vs 0.022 ± 0.002 in G_PFC_E, *p*=0.468, ANOVA, *n* = 8 mice for each group).

Brain-wide knock-down of DISC1 has been related to abnormal neuronal morphology and connectivity both during development (Chini et al., 2018) and at adulthood (Kvajo et al., 2008; Crabtree et al., 2017). To test whether these structural deficits are present also in the PFC of G_PFC_E mice, we undertook a detailed histological examination of the cytoarchitecture of tDimer-labeled pyramidal neurons in prefrontal layer II/III of P9 CON (*n* = 21 neurons from 3 mice), G_PFC_E (*n* = 21 neurons from 3 mice), and GE mice (*n* = 21 neurons from 3 mice). The complexity of dendritic branching of the tDimer-labeled neurons in layer II/III was assessed by Sholl analysis. Compared with CON, layer II/III pyramidal neurons of GE and G_PFC_E mice had significantly reduced dendritic branching (condition effect, *p* < 1 × 10−8, ANOVA; Fig. 1*F*). These deficits were particularly prominent within a radius of 20–70 μm from the cell soma center (*p* < 1 × 10^−6^ for all comparisons). Furthermore, similar to GE mice (164.78 ± 6.87 μm^2^, *p* = 0.38, ANOVA followed by Bonferroni-corrected *post hoc* test), G_PFC_E mice (181.40 ± 10.00 μm^2^) showed remarkable reduction (*p* < 1 × 10^−7^, ANOVA followed by Bonferroni-corrected *post hoc* test) in the soma size of layer II/III pyramidal neurons compared with CON mice (261.15 ± 9.29 μm^2^; Fig. 1*G*). Next, we examined the spine density along the dendrites of layer II/III pyramidal neurons in the three groups. Similar to GE mice (*n* = 21 neurons from 3 mice, 2.46 ± 0.08 per 10 μm, *p* = 0.44), G_PFC_E mice (*n* = 21 neurons from 3 mice, 2.19 ± 0.09 per 10 μm) had significantly lower spine density (*p* < 1 × 10^−9^, ANOVA followed by Bonferroni-corrected *post hoc* test) compared with CON mice (*n* = 20 neurons from 3 mice, 4.43 ± 0.26 per 10 μm; Fig. 1*H*). These data indicate that, similar to GE mice, G_PFC_E mice have a simplified dendritic arborization and decreased spine density.

**Figure 2.**
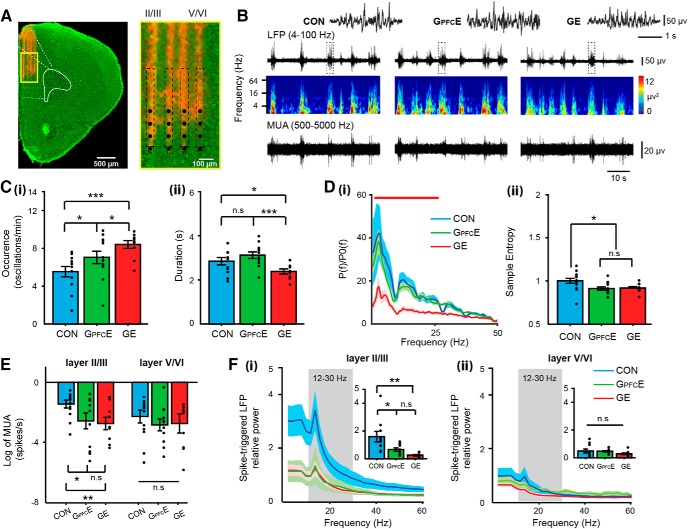
Patterns of oscillatory activity and neuronal firing in the PFC of neonatal G_PFC_E mice. ***A***, Digital photomontage reconstructing the location of the DiI-labeled 4 × 4-site recording electrode (orange) in a 100-μm-thick coronal section containing the PFC of a P9 mouse. Inset, The position of recording sites (black dots) over the prelimbic layers displayed at higher-magnification. ***B***, Extracellular LFP recording of discontinuous oscillatory activity in PL from a P9 CON (left), a P9 G_PFC_E (middle), and a P9 GE (right) mouse displayed after bandpass (4–100 Hz) filtering (top) and the corresponding MUA after bandpass (500–5000 Hz) filtering (bottom). Traces are accompanied by the color-coded wavelet spectra of the LFP at identical time scale. ***C***, Bar diagrams displaying the mean occurrence (***i***; one-way ANOVA: *p* = 0.0049, *F*_(2,31)_ = 6.34) and duration (***ii***; one-way ANOVA: *p* = 0.006, *F*_(2,31)_ = 6.06) of prefrontal oscillations recorded in CON, G_PFC_E, and GE mice. ***D***, ***Di***, Averaged power spectra *P*(*f*) of discontinuous oscillations normalized to the baseline power *P*0(*f*) of time windows lacking oscillatory activity. Red bar indicates significant difference between CON and GE mice (***p < 0.001). ***Dii***, Bar diagram displaying the mean sample entropy of prelimbic oscillations as a measure of the complexity of oscillatory activity recorded from CON, G_PFC_E, and GE mice (one-way ANOVA: *p* = 0.021, *F*_(2,31)_ = 4.41). ***E***, Bar diagram displaying the mean MUA of layer II/III and layer V/VI neurons in PFC of CON, G_PFC_E, and GE mice (one-way ANOVA, layer II/III: *p* = 0.038, *F*_(2,31)_ = 3.65; layer V/VI: *p* = 0.656, *F*_(2,30)_ = 0.428). ***F***, Power spectra of averaged spike-triggered LFP for layer II/III (***Fi***) and layer V/VI (***Fii***) of CON, G_PFC_E, and GE mice. Gray shadow highlights the 12–30 Hz frequency range. Insets, Bar diagrams displaying mean power values for the 12–30 Hz frequencies for spikes recorded in prelimbic layer II/III and V/VI, respectively, of CON, G_PFC_E, and GE mice (one-way ANOVA, layer II/III: *p* = 0.003, *F*_(2,25)_ = 7.61; layer V/VI: *p* = 0.27, *F*_(2,27)_ = 1.38). Data are presented as mean ± SEM. Significance levels of *p* > 0.05 (n.s.), *p* < 0.05 (*), *p* < 0.01 (**) and *p* < 0.001 (***) were detected.

Because DISC1 knock-down is spatially confined, G_PFC_E mice are instrumental for assessing the role of DISC1 for the functional development of prefrontal circuits. For this, we performed multisite extracellular recordings of LFP and MUA from the PL of P8–P10 urethane-anesthetized CON (*n* = 14), G_PFC_E (*n* = 13) and GE mice (*n* = 10). The four shanks of recording electrodes were confirmed to be located across layer II/III and V/VI of the PL (Fig. 2*A*). Our previous investigations revealed that network oscillations and neuronal firing have a similar structure and temporal organization in urethane-anesthetized and asleep non-anesthetized rodents of neonatal age (Bitzenhofer et al., 2015). Discontinuous (i.e., periods of network activity alternate with periods of “silence”) oscillatory discharges with frequency components peaking in theta (4–12 Hz) and β-low gamma frequency range (12–40 Hz) have been detected in all investigated mice (Fig. 2*B*,*D*). However, their properties differed between groups. In line with previous data (Hartung et al., 2016b), the prelimbic activity of GE mice appeared highly fragmented and correspondingly, the occurrence of oscillatory events was higher (8.40 ± 0.43 oscillations/min, *p* = 0.0002, ANOVA followed by Bonferroni-corrected *post hoc* test) and the duration shorter (2.38 ± 0.12 s, *p* = 0.016, ANOVA followed by Bonferroni-corrected *post hoc* test) compared with CON (5.53 ± 0.59 oscillations/min, 2.84 ± 0.18 s). The fragmented structure of discharges was present also in G_PFC_E mice, yet the occurrence increase was rather moderate (7.04 ± 0.68 oscillations/min, *p* = 0.045, ANOVA followed by Bonferroni-corrected *post hoc* test) and the duration of oscillatory events was unaffected (3.12 ± 0.15 s, *p* = 0.14, ANOVA followed by Bonferroni-corrected *post hoc* test; Fig. 2*C*). The relative power of oscillatory events normalized to the periods lacking coordinated activity was significantly decreased over all frequency bands in GE mice versus CON. In contrast, no differences were detected between CON and G_PFC_E mice (Fig. 2*Di*). Additionally, we analyzed the sample entropy of oscillatory events that reflects the complexity of developing neuronal networks (Kapucu et al., 2017). Compared with prefrontal oscillations in CON mice (1.01 ± 0.037), both GE (0.93 ± 0.02, *p* = 0.05, ANOVA followed by Bonferroni-corrected *post hoc* test) and G_PFC_E mice (0.92 ± 0.02, *p* = 0.028, ANOVA followed by Bonferroni-corrected *post hoc* test) had decreased sample entropy, suggesting that the structure of prelimbic circuits was less complex and most likely, more immature (Fig. 2*Dii*).

The abnormal temporal organization of coordinated activity in the PFC of GE and G_PFC_E mice led us to hypothesize that the local circuitry in the PL was similarly perturbed in the two groups of mice. To get further insights, we calculated the firing rates in layer II/III and layer V/VI of the two models (GE, *n* = 14; G_PFC_E, *n* = 13) and compared them with the values from CON (*n* = 10). Prelimbic neurons mostly fire during oscillatory events (Fig. 2*B*). Overall, DISC1 suppression caused significant MUA decrease in prelimbic layer II/III (CON: −1.45 ± 0.28; GE: −2.75 ± 0.44; G_PFC_E: −2.58 ± 0.55), yet no significant differences (*p* = 0.398, ANOVA followed by Bonferroni-corrected *post hoc* test) were detected between GE and G_PFC_E mice. The firing within layer V/VI was unchanged in all three mouse groups (CON: −2.29 ± 0.45; GE: −2.75 ± 0.67; G_PFC_E: −3.17 ± 0.42; Fig. 2*E*). Next, we aimed to deepen into the connectivity strength of local prefrontal circuits. For this, we calculated the spike-triggered power (STP) of the LFP. The method assesses the strength of postsynaptic activity at one cortical site caused by spiking at another location (Nauhaus et al., 2009; Ray and Maunsell, 2011). The 12–30 Hz power of relative STP within prelimbic layer II/III was significantly changed in GE (0.25 ± 0.06, *p* = 0.003, ANOVA followed by Bonferroni-corrected *post hoc* test) and G_PFC_E (0.66 ± 0.13, *p* = 0.028, ANOVA followed by Bonferroni-corrected *post hoc* test) compared with CON mice (1.59 ± 0.40; Fig. 2*Fi*). The coupling within deeper layers of PL was comparable in the three mouse groups (CON: 0.48 ± 0.16; GE: 0.28 ± 0.06; G_PFC_E: 0.46 ± 0.05, *p* = 0.269, ANOVA followed by Bonferroni-corrected *post hoc* test; Fig. 2*Fii*).

These data indicate that transient suppression of DISC1 in PFC causes sparser dendritic arborization and lower spine density, network deficits, and abnormal circuit wiring in the PL, which are similar to the dysfunction resulting from brain-wide DISC1 knock-down.

### Transient DISC1 knock-down confined to layer II/III pyramidal neurons in PFC does not perturb the firing and network activity in HP of neonatal immune-challenged mice

**Figure 3.**
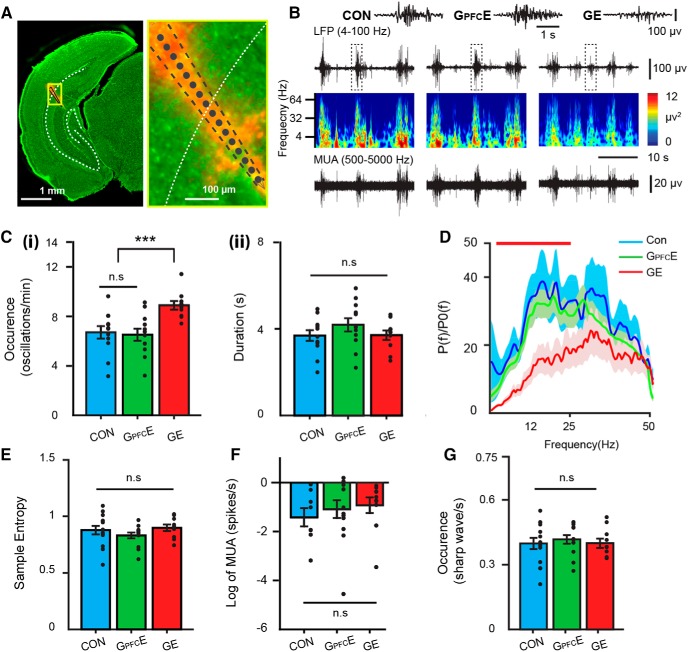
Patterns of oscillatory activity and neuronal firing in the CA1 area of intermediate/ventral HP of neonatal G_PFC_E mice. ***A***, Digital photomontage reconstructing the location of the DiI-labeled 1 × 16-site recording electrode (orange) in a 100-μm-thick coronal section containing the intermediate/ventral HP of a P9 mouse. Inset, The position of recording sites (gray dots) over the prelimbic layers displayed at higher-magnification. ***B***, Extracellular LFP recording of discontinuous oscillatory activity in PL from a P9 CON (left), a P9 G_PFC_E (middle), and a P9 GE (right) mouse displayed after bandpass (4–100 Hz) filtering (top) and the corresponding MUA after bandpass (500–5000 Hz) filtering (bottom). Traces are accompanied by the color-coded wavelet spectra of the LFP at identical time scale. ***C***, Bar diagrams displaying the mean occurrence (***Ci***; one-way ANOVA: *p* = 0.002, *F*_(2,31)_ = 7.63) and the duration (***Cii***; one-way ANOVA: *p* = 0.33, *F*_(2,31)_ = 1.16) of hippocampal oscillations recorded in CON, G_PFC_E, and GE mice. ***D***, Averaged power spectra *P*(*f*) of discontinuous oscillations normalized to the baseline power *P*0(*f*) of time windows lacking oscillatory activity. Red bar indicates significant difference between CON and GE mice. ***E***, Bar diagram displaying the mean sample entropy of hippocampal oscillations as measure of the complexity of oscillatory activity for CON, G_PFC_E, and GE mice (one-way ANOVA: *p* = 0.34, *F*_(2,34)_ = 1.13). ***F***, Bar diagram displaying the mean MUA of CA1 neurons in CON, G_PFC_E, and GE mice (one-way ANOVA: *p* = 0.66, *F*_(2,28)_ = 0.42). ***G***, Bar diagrams displaying the mean occurrence of SPW in CON, G_PFC_E, and GE mice (one-way ANOVA: *p* = 0.81, *F*_(2,34)_ = 0.22). Data are presented as mean ± SEM. Significance levels of *p* > 0.05 (n.s.), and *p* < 0.001 (***) were detected.

Previous data identified the CA1 area in intermediate/ventral HP as major monosynaptic drive for the oscillatory entrainment of prelimbic circuits during development (Brockmann et al., 2011; Ahlbeck et al., 2018). The activation of prelimbic circuits impacts HP via subcortical relay stations, such as midline thalamus, but not via direct axonal projections (Hartung et al., 2016a). To assess the effects of DISC1 suppression on hippocampal activity, we compared the oscillatory patterns and neuronal firing in the CA1 area of CON (*n* = 14), GE (*n* = 13), and G_PFC_E (*n* = 10) mice (Fig. 3*A*). In line with previous data (Hartung et al., 2016b), the discontinuous oscillatory activity of HP with frequencies within theta-β ranges (Fig. 3*B*,*D*) was changed by the combination of maternal immune activation with brain-wide suppression of DISC1 function. The occurrence (8.88 ± 0.37 oscillations/min) of oscillations (4–100 Hz) in GE mice was significantly increased (*p* = 0.001, ANOVA followed by Bonferroni-corrected *post hoc* test), whereas their relative power, especially in theta (4–12 Hz) frequency (7.10 ± 1.56), was significantly (*p* = 0.024, ANOVA followed by Bonferroni-corrected *post hoc* test) decreased compared with the HP activity of CON mice (occurrence: 6.70 ± 0.52 oscillations/min; relative power: 20.06 ± 4.56). The duration of oscillatory events and their complexity mirrored by sample entropy were similar in GE (duration: 3.71 ± 0.23; sample entropy: 0.90 ± 0.03) and CON (duration: 3.69 ± 0.26; sample entropy: 0.88 ± 0.04) mice. The transient prefrontal-restricted suppression of DISC1 did not affect the properties of oscillatory events in G_PFC_E mice. The occurrence (6.50 ± 0.51 oscillations/min), duration (4.19 ± 0.32 s), relative power (4–12 Hz: 17.10 ± 1.92), and sample entropy (0.83 ± 0.3) were similar to the values of CON mice. Moreover, the firing rate of HP neurons (−1.08 ± 0.38) or the occurrence of sharp waves (SPWs; 0.42 ± 0.02 /s) was comparable in G_PFC_E, GE (firing rate: −0.93 ± 0.33; SPW occurrence: 0.40 ± 0.02 /s), and CON mice (firing rate: −1.42 ± 0.40, *p* = 0.66, ANOVA followed by Bonferroni-corrected *post hoc* test; SPW occurrence: 0.40 ± 0.03 /s, *p* = 0.81, ANOVA followed by Bonferroni-corrected *post hoc* test; Fig. 3*F*,*G*).

These data indicate that, in contrast to brain-wide knock-down of DISC1, transient suppression of DISC1 in PFC does not perturb the firing and network activity in intermediate/ventral HP of immune-challenged mice at neonatal age.

### Transient DISC1 knock-down confined to layer II/III pyramidal neurons in PFC causes weaker long-range coupling in neonatal immune-challenged mice

**Figure 4.**
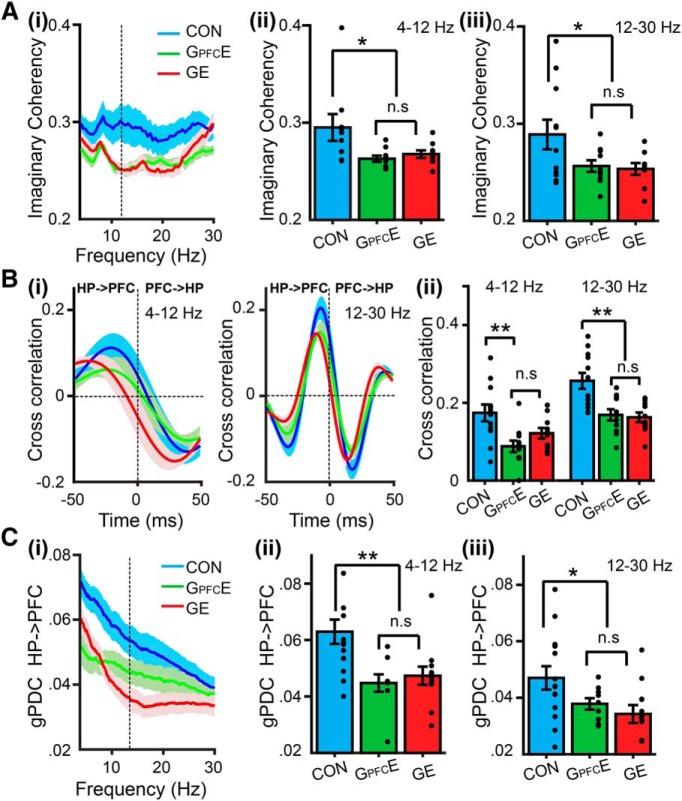
Coupling by synchrony and directed interactions within prefrontal–hippocampal networks of neonatal G_PFC_E mice. ***Ai***, Mean coherence spectra for oscillatory activity simultaneously recorded in PL and hippocampal CA1 area of CON, G_PFC_E, and GE mice. ***Aii***, Bar diagram displaying the imaginary coherency when averaged for 4–12 Hz band in CON, G_PFC_E, and GE mice (one-way ANOVA: *p* = 0.037, *F*_(2,27)_ = 3.73). ***Aiii***, Same as ***Aii***, for 12–30 Hz in CON, G_PFC_E, and GE mice (one-way ANOVA: *p* = 0.047, *F*_(2,27)_ = 3.43). ***Bi***, Plot of cross-correlation of prelimbic and hippocampal oscillations within 4–12 Hz (left) and 12–30 Hz (right) when averaged for all investigated CON, G_PFC_E, and GE mice. Negative time lags correspond to HP leading PFC. ***Bii***, Bar diagram displaying the mean peak cross-correlation when averaged for 4–12 Hz in CON, G_PFC_E, and GE mice (one-way ANOVA: *p* = 0.005, *F*_(2,30)_ = 6.36) and 12–30 Hz bands in CON, G_PFC_E, and GE mice (one-way ANOVA: *p* = 0.0000, *F*_(2,30)_ = 10.09). ***Ci***, Plot of mean gPDC in relationship to frequency for HP → PL in CON, G_PFC_E, and GE mice. ***Cii***, Bar diagram displaying gPDC when averaged for 4–12 Hz in CON, G_PFC_E, and GE mice (one-way ANOVA: *p* = 0.003, *F*_(2,32)_ = 6.97). ***Ciii***, Same as ***Cii*** for at 12–30 Hz in CON, G_PFC_E, and GE mice (one-way ANOVA: *p* = 0.034, *F*_(2,32)_ = 3.78). Data are presented as mean ± SEM. Significance levels of *p* < 0.05 (n.s.), *p* < 0.05 (*) and *p* < 0.01 (**) were detected.

Several analytical approaches were used to test whether the transient suppression of DISC1 confined to PFC affects the coupling between PL and HP. First, we calculated the imaginary part of coherency between PL and HP of CON, G_PFC_E, and GE mice. The method has been described to be insensitive to spurious connectivity arising from volume conduction (Nolte et al., 2004). Consistent with previous data (Hartung et al., 2016b), the tight coupling within prefrontal–hippocampal networks of neonatal CON mice was profoundly altered in GE mice (Fig. 4*A*). Brain-wide suppression of DISC1 function caused a significant decrease of prefrontal–hippocampal coherency within 4–12 Hz (0.268 ± 0.004, *p* = 0.040, ANOVA followed by Bonferroni-corrected *post hoc* test) and 12–30 Hz ranges (0.254 ± 0.006, *p* = 0.025, ANOVA followed by Bonferroni-corrected *post hoc* test) compared with CON mice (4–12 Hz: 0.295 ± 0.014; 12–30 Hz: 0.289 ± 0.016). Similar coupling decrease was observed when the DISC1 suppression was confined to PFC. The prefrontal–hippocampal coherency in G_PFC_E mice was similar with that of GE mice, but weaker both within 4–12 Hz (0.263 ± 0.003, *p* = 0.022, ANOVA followed by Bonferroni-corrected *post hoc* test) and 12–30 Hz range (0.257 ± 0.006, *p* = 0.035, ANOVA followed by Bonferroni-corrected *post hoc* test) compared with the values calculated for CON mice (4–12 Hz: 0.295 ± 0.014; 12–30 Hz: 0.289 ± 0.016; Fig. 4*A*).

Second, we assessed the directionality of interactions between PL and HP in the three groups of mice by calculating time-resolved cross-correlation and frequency-resolved gPDC. In line with previous results (Hartung et al., 2016b), max cross-correlation of 4–12 and 12–30 Hz oscillations within prefrontal–hippocampal networks of all investigated mice was detected for HP → PL (Fig. 4*B*), yet the magnitude of the hippocampal drive differed between the groups. Suppression of DISC1 in PFC of G_PFC_E mice led to cross-correlation values (4–12 Hz: 0.09 ± 0.002; 12–30 Hz: 0.17 ± 0.002) similar to those of GE mice (4–12 Hz: 0.12 ± 0.01, *p*=0.06, ANOVA followed by Bonferroni-corrected *post hoc* test; 12–30 Hz: 0.16 ± 0.01, *p* = 0.37, ANOVA followed by Bonferroni-corrected *post hoc* test), but significantly decreased compared with those calculated for CON mice (4–12 Hz: 0.17 ± 0.02, *p* = 0.002, ANOVA followed by Bonferroni-corrected *post hoc* test; 12–30 Hz: 0.26 ± 0.02, *p* = 0.001, ANOVA followed by Bonferroni-corrected *post hoc* test). Next, we calculated the gPDC between the PL and HP, a measure that reflects the directionality of network interactions in different frequency bands (Fig. 4*C*). Both brain-wide suppression of DISC1 function and the transient prefrontal-restricted suppression of DISC1 caused a decreased drive from HP to PL within 4–12 Hz (GE: 0.047 ± 0.003, *p* = 0.004, ANOVA followed by Bonferroni-corrected *post hoc* test; G_PFC_E: 0.045 ± 0.003, *p* = 0.001, ANOVA followed by Bonferroni-corrected *post hoc* test) and 12–30 Hz (GE: 0.034 ± 0.003, *p* = 0.011, ANOVA followed by Bonferroni-corrected *post hoc* test; G_PFC_E: 0.038 ± 0.002, *p* = 0.031, ANOVA followed by Bonferroni-corrected *post hoc* test) compared with CON mice (4–12 Hz: 0.063 ± 0.004; 12–30 Hz: 0.047 ± 0.004).

These data indicate that transient suppression of DISC1 restricted to PFC during neonatal development causes weaker long-range prefrontal–hippocampal coupling that is similar to the dysfunction resulting from brain-wide DISC1 knock-down. Because the hippocampal activity of G_PFC_E mice is normal (Fig. 3), the decreased coupling between PFC and HP after transient suppression of DISC1 in PFC most likely mirrors the poorer ability of locally disrupted prefrontal circuits to follow the hippocampal drive.

### Transient prefrontal DISC1 knock-down causes poorer recognition memory performance of pre-juvenile immune-challenged mice

**Figure 5.**
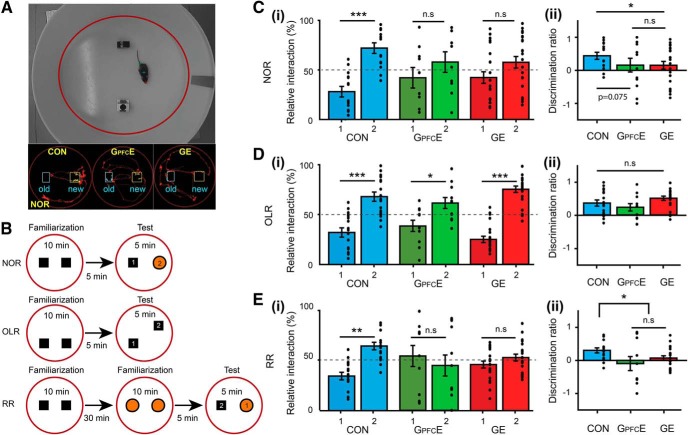
Novelty recognition of pre-juvenile G_PFC_E mice. ***A***, Top, Photograph of the arena used for NOR, OLR, and RR. Bottom, Representative tracking images illustrating test trials for the NOR test performed by a P17 CON (left), a P17 G_PFC_E (middle), and a P17 GE mouse (right). The computer generated track of the mouse pup (red) is displayed together with zones (blue yellow) created around the objects. ***B***, Schematic diagrams of the protocol for NOR, OLR and RR tasks. ***Ci***, Bar diagram illustrating the relative interaction time spent by CON, G_PFC_E, and GE with the objects during the NOR test trial. The dotted line indicates chance level. ***Cii***, Bar diagram displaying the mean discrimination ratio when averaged for CON, G_PFC_E, and GE mice during NOR task in CON, G_PFC_E, and GE mice (one-way ANOVA: *p* = 0.049, *F*_(2,48)_ = 3.18). ***Di–Dii***, ***Ei–Eii***, Same as ***Ci–Cii*** for CON, G_PFC_E, and GE mice in the OLR (one-way ANOVA: *p* = 0.09, *F*_(2,47)_ = 2.43) and RR (one-way ANOVA: *p* = 0.034, *F*_(2,45)_ = 3.66) test trial, respectively. Data are presented as mean ± SEM. Significance levels of *p* > 0.05 (n.s.), *p* < 0.05 (*), *p* < 0.01 (**) and *p* < 0.001 (***) were detected.

A major question that needs to be addressed is whether transient suppression of DISC1 in neonatal PFC perturbs the network function throughout development and consequently, the related cognitive performance later in life. We recently showed that cognitive abilities that rely on prefrontal–hippocampal coupling and emerge at pre-juvenile age (i.e., P17–P20) are impaired when brain-wide DISC1 knock-down was combined with prenatal immune challenge (Hartung et al., 2016b). Here, we compare the behavioral performance of G_PFC_E mice with that of CON and GE mice to elucidate the long-term impact of transient DISC1 knock-down confined to layer II/III of PFC. For this, we monitored the novelty detection and recognition memory, which have been shown to result from interactions between PFC and HP (Warburton and Brown, 2015). These abilities can be easily tested at pre-juvenile age because they rely on the mouse's intrinsic exploratory drive and require no prior training or deprivation (Krüger et al., 2012). Specifically, we tested novel object recognition (NOR), OLR, and RR in CON (*n* = 17), GE (*n* = 23), and G_PFC_E (*n* = 12) mice using a custom-designed arena and previously established protocols (Fig. 5*A*,*B*). During the familiarization trials of these tests, all mice spent equal time investigating the two objects placed in the arena. During the NOR test trial, CON mice spent significantly longer time interacting with the novel object (71.97 ± 5.55%, *t*_(16)_ = −4.11, *p* = 0.0006, pared *t* test) than with the familiar one (28.03 ± 5.55%). In contrast, GE mice failed to distinguish between the two objects (familiar: 46.44 ± 7.98%; novel: 53.56 ± 7.98%, *t*_(22)_ = −1.32, *p* = 0.325, pared *t* test). Similarly, pre-juvenile G_PFC_E mice were also unable to distinguish between the two objects during test trial (familiar: 42.09 ± 10.83%; novel: 57.91 ± 10.83%, *t*_(11)_ = −0.76, *p* = 0.231, pared *t* test; Fig. 5*C*). During the OLR test trial, all mice spent more time to explore the relocated object (CON: 67.99 ± 5.48%, *t*_(16)_ = −3.84, *p* = 0.002, pared *t* test; G_PFC_E: 61.38 ± 5.81%, *t*_(11)_ = −2.06, *p* = 0.033, pared *t* test; GE: 73.28 ± 4.57%, *t*_(22)_ = −7.08, *p* < 1 × 10^−7^, pared *t* test) than the object with constant position (CON: 32.01 ± 5.48%; G_PFC_E: 38.62 ± 5.81%; GE: 26.72 ± 4.57%; Fig. 5*D*). The similar discrimination ratio (CON: 0.36 ± 0.11; GE: 0.47 ± 0.09, *p* = 0.225, ANOVA followed by Bonferroni-corrected *post hoc* test; G_PFC_E: 0.23 ± 0.12, *p* = 0.199, ANOVA followed by Bonferroni-corrected *post hoc* test; Fig. 5*Dii*) indicates that the OLR was intact in all investigated mice. During RR task, mice needed to process temporal information by recognizing the object with which they most recently interacted (Fig. 5*E*). The CON mice spent more time with the object they explored during the first familiarization trial than the new object from the second familiarization trial (old: 66.43 ± 4.41%, recent: 33.57 ± 4.41%, *t*_(16)_ = −3.96, *p* = 0.0009, pared *t* test). However, both G_PFC_E and GE mice failed to recognize the most recently explored object and spent equal time with both objects (GE, old: 47.53 ± 3.49%, recent: 52.47 ± 3.49%, *t*_(22)_ = −1.02, *p* = 0.238, pared *t* test; G_PFC_E, old: 45.21 ± 11.06%, recent: 54.79 ± 11.06%, *t*_(11)_ = −0.45, *p* = 0.330, pared *t* test). Correspondingly, the discrimination ratio between the old and the recent object significantly decreased (GE: −0.10 ± 0.22, *p* = 0.0009, ANOVA followed by Bonferroni-corrected *post hoc* test; G_PFC_E: −0.05 ± 0.07, *p* = 0.042, ANOVA followed by Bonferroni-corrected *post hoc* test) compared with the values for CON mice (0.33 ± 0.09).

The incapacity to perform NOR and RR tasks may result from poor motor abilities and/or enhanced anxiety when interacting with the objects. To test this hypothesis, we first analyzed the exploratory behavior of P16 CON, GE, and G_PFC_E mice. The distance covered was similar in all groups (CON: 1242 ± 159 cm; GE: 1032 ± 123 cm; G_PFC_E: 1030 ± 154 cm, *p* = 0.11, ANOVA followed by Bonferroni-corrected *post hoc* test). Moreover all mice spent more time in the outer circle than in the inner circle of the arena (CON: 1127 ± 132 cm vs 149 ± 32 cm; GE: 958 ± 111 cm vs 73 ± 18 cm; G_PFC_E: 941 ± 139 cm vs 89 ± 33 cm) and had similar latencies when entering the inner circle (CON: 73.85 ± 24.45 s, GE: 101.83 ± 30.44 s, G_PFC_E: 92.29 ± 48.83 s). These results suggest that exploratory and anxiety abilities were similar in CON, GE, and G_PFC_E mice.

Thus, transient prefrontal DISC1 knock-down has long-lasting behavioral effects, being sufficient to impair novel object and recency recognition in immune-challenged mice at pre-juvenile age.

### Transient prefrontal DISC1 knock-down causes weaker prefrontal–hippocampal coupling throughout development in immune-challenged mice

**Figure 6.**
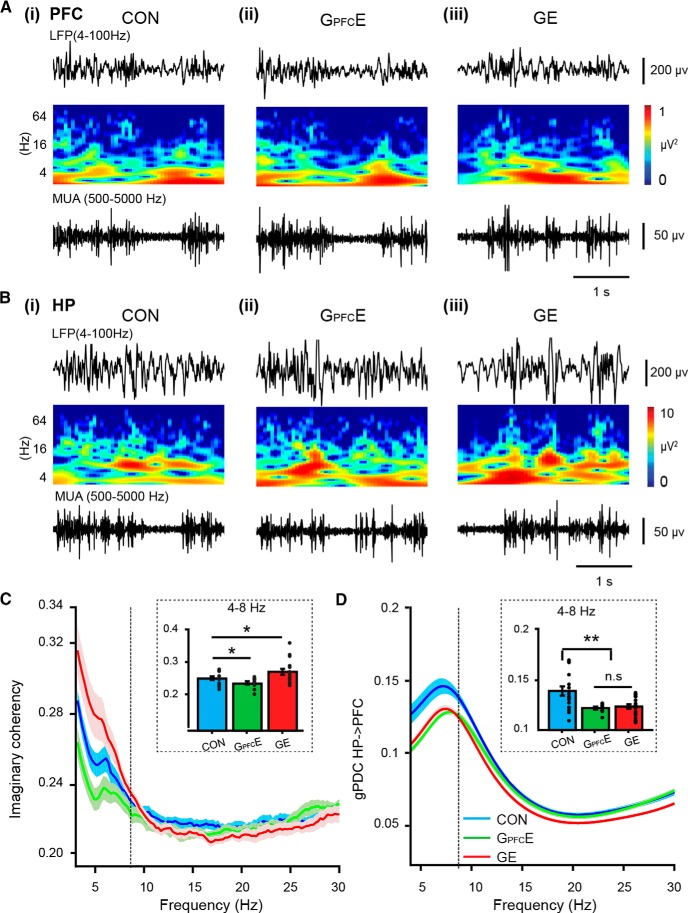
Activity patterns and coupling by synchrony within prefrontal–hippocampal networks of pre-juvenile G_PFC_E mice. ***Ai***, Extracellular LFP recording of continuous oscillatory activity in PL from a P22 CON mouse displayed after bandpass (4–100 Hz) filtering (top) and the corresponding MUA after bandpass (500–5000 Hz) filtering (bottom). Traces are accompanied by the color-coded wavelet spectra of the LFP at identical time scale (middle). ***Aii***, Same as ***Ai*** for a P22 G_PFC_E mice. ***Aiii***, Same as ***Ai*** for a P22 GE mice. ***Bi–Biii***, Same as ***Ai–Aiii*** for HP, respectively. ***C***, Mean coherence spectra for oscillatory activity simultaneously recorded in PL and hippocampal CA1 area of CON, G_PFC_E, and GE mice. Inset, Bar diagram displaying the mean imaginary part of coherence when averaged for each group of pups (one-way ANOVA: *p* = 0.013, *F*_(2,37)_ = 4.94). ***D***, Plot of mean gPDC in relationship to frequency for HP → PL in CON, G_PFC_E, and GE mice. Inset, Bar diagram displaying gPDC when averaged for 4–8 Hz (one-way ANOVA: *p* = 0.021, *F*_(2,37)_ = 4.30). Data are presented as mean ± SEM. Significance levels of *p* > 0.05 (n.s.), *p* < 0.05 (*), *p* < 0.01 (**) and were detected.

To assess the mechanisms underlying the behavioral deficits in pre-juvenile G_PFC_E mice, we tested the hypothesis that transient suppression of DISC1 confined to PFC permanently perturbs the maturation of prefrontal–hippocampal circuits. As a readout of perturbation we used the oscillatory patterns and neuronal firing of pre-juvenile PL and CA1 area of intermediate/ventral HP, as well as their coupling by synchrony. For this, we performed multisite extracellular recordings of LFP and MUA simultaneously from both areas of urethane-anesthetized P20–P23 mice (CON, *n* = 14; G_PFC_E, *n* = 10; GE, *n* = 16). As previously reported (Hartung et al., 2016b); all investigated mice showed similar patterns of network activity, which correspond to the sleep-like rhythms mimicked by urethane anesthesia (Wolansky et al., 2006; Clement et al., 2008; Pagliardini et al., 2013; Fig. 6*A*,*B*). Continuous large-amplitude slow oscillations were superimposed with oscillatory activity in faster theta (4–12 Hz) and gamma (30–100 Hz) frequencies. The amplitude and power of these prelimbic and hippocampal oscillatory patterns were similar in CON, GE, and G_PFC_E mice (Table 1; Fig. 6*A*,*B*). Lower firing rates in layer II/III were detected in GE, but not G_PFC_E mice. In contrast, significant changes in the prelimbic–hippocampal coupling within 4–8 Hz have been detected (Fig. 6*C*). In line with our previous results (Hartung et al., 2016b), the synchrony between PL and HP mirrored by the imaginary part of the coherency for 4–8 Hz range was augmented in GE mice (0.273 ± 0.011, *p* = 0.029, ANOVA followed by Bonferroni-corrected *post hoc* test) compared with CON mice (0.249 ± 0.006). Transient DISC1 suppression in PFC had an opposite effect, the theta band imaginary coherency in G_PFC_E mice was significantly decreased (0.235 ± 0.005, *p* = 0.046, ANOVA followed by Bonferroni-corrected *post hoc* test) compared with CON mice. To investigate whether the directionality of interactions between PL and HP was affected by transient DISC1 suppression in PFC, we quantified the theta band drive from HP to PFC by gPDC (Fig. 6*D*). Both GE and G_PFC_E mice showed decreased causal interactions from HP to PL within 4–8 Hz (GE: 0.115 ± 0.003, *p* = 0.002, ANOVA followed by Bonferroni-corrected *post hoc* test; G_PFC_E: 0.116 ± 0.002, *p* = 0.049, ANOVA followed by Bonferroni-corrected *post hoc* test) compared with CON mice (0.126 ± 0.004).

**Table 1.** Properties of continuous oscillatory activity and neuronal firing in PL and HP of pre-juvenile CON, GE, and G_PFC_E mice

	PL			HP		
	CON	GE	G_PFCE_	CON	GE	G_PFCE_
Power 4–12 Hz, dB μV^2^/Hz	180.58 ± 12.89	205.99 ± 15.10	188.02 ± 23.05	269.23 ± 18.76	299.34 ± 25.40	241.31 ± 22.21
Power 12–30 Hz, dB μV^2^/Hz	20.34 ± 1.41	22.92 ± 2.22	20.18 ± 1.31	45.78 ± 3.26	44.33 ± 3.51	41.90 ± 2.11
Power 30–100 Hz, dB μV^2^/Hz	3.32 ± 0.24	3.38 ± 0.30	3.71 ± 0.28	9.14 ± 0.53	8.78 ± 0.45	10.22 ± 0.68
MUA, spikes/s	Layer II/III: 2.07 ± 0.14	Layer II/III: 1.45 ± 0.10	Layer II/III: 2.03 ± 0.14	1.64 ± 0.11	1.20 ± 0.15	1.50 ± 0.12
		***p = 0.0005			**p = 0.011	
	Layer V/VI: 1.71 ± 0.16	Layer V/VI: 1.55 ± 0.18	Layer V/VI: 1.63 ± 0.17			

Data are shown as mean ± SEM. Significance was assessed using one-way ANOVA and the listed values correspond to comparisons between CON and GE mice. Data are presented as mean ± SEM.

Significance levels of *p* < 0.01 (**) and *p* < 0.001 (***) were detected.

These results indicate that transient prefrontal DISC1 knock-down during neonatal development permanently impairs the long-range coupling between PL and HP, but the changes are less prominent than in GE mice.

## Discussion

Neuronal network assembly during development is controlled by numerous genetic and environmental factors. The maturation of prefrontal–hippocampal circuits has been shown to be shaped by both DISC1, as molecular hub of multiple developmental processes, and prenatal immune challenge (Hartung et al., 2016b). In the present study, we combined multisite electrophysiological recordings *in vivo*, neuroanatomy and behavioral investigation of CON, G_PFC_E, and GE mice and provide evidence that (1) confinement of DISC1 suppression to perinatal PFC by *in utero* gene transfer leads to abnormal prefrontal network activity and neuronal firing in neonatal mice experiencing a prenatal immune challenge, which results from structural and functional deficits of layer II/III pyramidal neurons; (2) the prefrontal dysfunction of neonatal G_PFC_E mice is largely similar to that described for GE mice (Table 2); (3) coupling by synchrony and directed interactions between PFC and HP are weaker, yet the HP activity is normal in G_PFC_E mice; and (4) transient DISC1 suppression in neonatal PFC of immune-challenged mice is sufficient to disrupt the communication within prefrontal–hippocampal networks throughout neonatal and pre-juvenile development and to impair the behavioral performance of juvenile mice in recognition memory tasks. These results uncover the consequences of transient DISC1 suppression throughout development and highlight the critical relevance of pyramidal neurons in layer II/III for local circuit wiring. They complement previous findings on the abnormal information processing and cognitive performance of adult mice (Niwa et al., 2010).

**Table 2.** Summary results show that transient suppression of DISC1 in PFC causes abnormal morphology, network activity in PFC, and weaker long-range PFC–HP coupling, whereas the hippocampal activity is normal

	GE vs CON	G_PFC_E vs CON	G_PFC_E vs GE
PFC			
Dendrite branching	↓	↓	—
Soma size	↓	↓	—
Spine density	↓	↓	—
Oscillatory event			
Occurrence	↑	↑	↓
Duration	↓	—	↓
Power			
4–12 Hz	↓	—	↓
12–30 Hz	↓	—	↓
Sample entropy	↓	↓	—
MUA	↓	↓	—
Spike triggered LFP	↓	↓	—
PFC–HP coupling			
Coherence			
4–12 Hz	↓	↓	—
12–30 Hz	↓	↓	—
Cross-correlation			
4–12 Hz	↓	↓	—
12–30 Hz	↓	↓	—
gPDC			
4–12 Hz	↓	↓	—
12–30 Hz	↓	↓	—
HP			
Oscillatory event			
Occurrence	↓	—	↓
Duration	—	—	—
Power			
4–12 Hz	↓	—	↑
12–30 Hz	↓	—	↑

↓, Significant decrease; ↑, significant increase; —, no change.

In line with previous data maternal immune activation (i.e., environmental stressor) or brain-wide DISC1 dysfunction alone had almost no impact on the prefrontal–hippocampal function at neonatal age (Hartung et al., 2016b). Therefore, the similar dysfunction observed in GE and G_PFC_E mice of this age supports the central role of developmental DISC1-controlled processes in PFC for the maturation of limbic circuits. As intracellular hub, DISC1 interacts with a large number of synaptic and cytoskeletal molecules. By these means, DISC1 controls synaptic plasticity processes in the adult brain (Greenhill et al., 2015; Tropea et al., 2018). Moreover, DISC1 interferes with neuronal proliferation and migration as well as with neurite outgrowth, formation, and maintenance of synapses (Brandon, 2007). Suppression of DISC1 has been reported to decrease spine density and impaired neurite outgrowth through disorganized microtubule-associated dynein motor complex (Ozeki et al., 2003; Kamiya et al., 2005). These morphological deficits have been observed in neonatal GE mice (Chini et al., 2018) and adult mice with brain-wide DISC1 knock-down (Kvajo et al., 2008; Crabtree et al., 2017). In G_PFC_E mice these structural deficits are likely to be confined to layer II/III pyramidal neurons in PFC. As a result, the firing rate and timing of these cells to network oscillations were significantly disrupted, whereas the overall network activity was mildly impaired compared with GE mice. The lack of effects on oscillatory power might be additionally due to the fact that the *in utero* gene transfer causes DISC1 knock-down in only one-third of layer II/III pyramidal neurons (Bitzenhofer et al., 2017b). The abnormal firing of layer II/III pyramidal neurons in PFC was sufficient to perturb the long-range coupling with HP, yet the oscillatory activity and neuronal firing in CA1 area of HP were similar to those of control pups. The decreased prefrontal spiking timed at β frequencies caused desynchronized entrainment of PFC in neonatal G_PFC_E mice. We suggest that the HP drive, even if not compromised by the local DISC1 suppression, cannot induce network activation, because of decreased connectivity and sparse synaptic transmission of layer II/III pyramidal neurons. Our previous investigations have shown that these neurons are key players for the emergence of β-gamma activity in the neonatal PFC in the presence of the excitatory drive from CA1 area (Bitzenhofer et al., 2017b; Ahlbeck et al., 2018).

Even if DISC1 suppression in PFC is transient and the DISC1 expression recovers to control level during pre-juvenile period, the effects of transient knock-down persist throughout development. Disruption of DISC1 for a maximum of 48 h has been reported to permanently affect the synaptic transmission within cortical circuits as result of underdeveloped dendritic arborization and reduced spine activity (Greenhill et al., 2015). It is very likely that the aberrant morphology of layer II/III pyramidal neurons during neonatal and pre-juvenile development causes abnormal interactions with interneurons and consequently, miswiring of local circuitry in PFC. DISC1 suppression indirectly perturbs the interneuronal function in adults (Cardarelli et al., 2018). Moreover, DISC1 interferes with immune-relevant signaling pathways early in life (Beurel et al., 2010). The structural and functional deficits caused by the combination of DISC1 suppression with MIA might persist and even augment throughout the life span, leading to altered cognitive and social behavior (Abazyan et al., 2010; Ibi et al., 2010; Lipina et al., 2013). Indeed, the weaker coupling through synchrony within prefrontal–hippocampal circuits in G_PFC_E mice persisted at pre-juvenile age, although the frequency-distribution and power of continuous oscillatory rhythms in both areas are unchanged compared with controls. In contrast, brain-wide DISC1 knock-down has the opposite effect, an exaggerated prefrontal–hippocampal coupling being detected. This effect may result from attempts to compensate the profoundly brain-wide miswiring.

In line with the long-lasting dysfunction of prefrontal–hippocampal coupling, behavioral abilities relying on this circuit were impaired in G_PFC_E mice. Both the ability to recognize novel objects and their recency were absent in G_PFC_E mice, whereas the recognition of new location was similar to that of CON mice. A widely accepted model identified prefrontal–hippocampal coupling as a crucial factor for novel object and recency recognition (Barker and Warburton, 2011).

These findings demonstrate that the development of PFC has a critical relevance for pathophysiological processes related to mental disorders. Abnormal DISC1 has been proposed to augment the risk of schizophrenia, bipolar disorders, and recurrent major depression (Kirkpatrick et al., 2006; Carlisle et al., 2011), especially when combined with environmental stressors acting at different developmental time points (van Os and Kapur, 2009; Insel, 2010). The present results offer mechanistic developmental explanations of structural, functional, and behavioral deficits observed at adulthood. The abnormal timing of layer II/III pyramidal neurons in relationship with the discontinuous neonatal oscillatory activity when DISC1 was selectively knocked-down in PFC leads to a persistent disturbance of long-range coupling within prefrontal–hippocampal circuits throughout development and finally, to poorer behavioral performance. Schizophrenia patients show decreased arborization and synaptic deficits in layer II/III pyramidal neurons, as well as alterations in parvalbumin-positive interneurons (Selemon and Goldman-Rakic, 1999; Lewis et al., 2005). Moreover, the prefrontal–hippocampal coupling is profoundly perturbed and the coactivation of the two brain areas weaker in schizophrenia (Meyer-Lindenberg et al., 2001). The present results support the neurodevelopmental origin of schizophrenia and related disorders and highlight the relevance of prefrontal processes during early maturation for the functional and cognitive deficits later in life.
